# GVCBLUP: a computer package for genomic prediction and variance component estimation of additive and dominance effects

**DOI:** 10.1186/1471-2105-15-270

**Published:** 2014-08-09

**Authors:** Chunkao Wang, Dzianis Prakapenka, Shengwen Wang, Sujata Pulugurta, Hakizumwami Birali Runesha, Yang Da

**Affiliations:** Department of Animal Science, University of Minnesota, Saint Paul, MN 55108 USA; Research Computing Center, The University of Chicago, Chicago, IL 60637 USA

**Keywords:** GVCBLUP, Genomic selection, Variance component, Heritability, BLUP

## Abstract

**Background:**

Dominance effect may play an important role in genetic variation of complex traits. Full featured and easy-to-use computing tools for genomic prediction and variance component estimation of additive and dominance effects using genome-wide single nucleotide polymorphism (SNP) markers are necessary to understand dominance contribution to a complex trait and to utilize dominance for selecting individuals with favorable genetic potential.

**Results:**

The GVCBLUP package is a shared memory parallel computing tool for genomic prediction and variance component estimation of additive and dominance effects using genome-wide SNP markers. This package currently has three main programs (GREML_CE, GREML_QM, and GCORRMX) and a graphical user interface (GUI) that integrates the three main programs with an existing program for the graphical viewing of SNP additive and dominance effects (GVCeasy). The GREML_CE and GREML_QM programs offer complementary computing advantages with identical results for genomic prediction of breeding values, dominance deviations and genotypic values, and for genomic estimation of additive and dominance variances and heritabilities using a combination of expectation-maximization (EM) algorithm and average information restricted maximum likelihood (AI-REML) algorithm. GREML_CE is designed for large numbers of SNP markers and GREML_QM for large numbers of individuals. Test results showed that GREML_CE could analyze 50,000 individuals with 400 K SNP markers and GREML_QM could analyze 100,000 individuals with 50K SNP markers. GCORRMX calculates genomic additive and dominance relationship matrices using SNP markers. GVCeasy is the GUI for GVCBLUP integrated with an existing software tool for the graphical viewing of SNP effects and a function for editing the parameter files for the three main programs.

**Conclusion:**

The GVCBLUP package is a powerful and versatile computing tool for assessing the type and magnitude of genetic effects affecting a phenotype by estimating whole-genome additive and dominance heritabilities, for genomic prediction of breeding values, dominance deviations and genotypic values, for calculating genomic relationships, and for research and education in genomic prediction and estimation.

**Electronic supplementary material:**

The online version of this article (doi:10.1186/1471-2105-15-270) contains supplementary material, which is available to authorized users.

## Background

Genomic prediction using genome-wide single nucleotide polymorphism (SNP) has become a powerful approach to capture genetic effects dispersed over the genome for predicting an individual’s genetic potential of a phenotype [1–3]. Genomic estimation of variance components using genome-wide SNP markers is a powerful tool for estimating the genetic contribution of the whole-genome to a phenotype and for addressing the missing heritability problem where a large number of causal variants explained only a small fraction of the phenotypic variation. Dominance effects of quantitative traits are measured as the deviation of the mean value of the heterozygote genotype of individuals from the averages of the two alternative homozygous genotypes [4, 5]. The inclusion of dominance in the prediction model may improve the accuracy of genomic prediction when dominance effects are present [6–9]. However, currently available software packages for genomic prediction and variance component estimation either are designed for additive effects only (GCTA [10]), or require users to prepare a dominance-specific file to estimate dominance effects (BLR or BGLR [11], GenSel [12], DMU [13], BLUPF90 [14]). User-friendliness of the computing tool affects the efficiency of data analysis for genomic prediction and estimation. In order to fill these gaps, we implement two computationally complementary computing strategies with identical results and various definitions of genomic relationships in the GVCBLUP package that has a wide-range of flexibility and functionality for broad applicability of genomic prediction and estimation of additive and dominance effects.

## Implementation

GVCBLUP currently has three main programs and a graphical user interface (GUI) named GVCeasy that integrates the three main programs with an existing program for graphical viewing of SNP effects. The three main programs are GREML_CE, GREML_QM, and GCORRMX, which are developed using shared memory parallel computing technology. GVCeasy supplies users a user-friendly platform to run GVCBLUP.

### Two complementary computing strategies

Two sets of formulations with complementary computing advantages and identical results based on two equivalent mixed models are implemented: the CE set for large numbers of SNP markers and the QM set for large numbers of individuals [5, 15]. Using notations in [5], the mixed model and its variance-covariance matrix for the CE set of formulations are:
12

where **X** = N × c model matrix for fixed non-genetic effects, **b** = c × 1 column vector of fixed effects, **Z** = N × q model matrix allocating phenotypic observations to SNP marker genotypes of individuals, **T**_α_ = q × m normalized model matrix for gene substitution effects of SNP markers, **α** = m × 1 column vector of gene substitution effects of SNP markers, **T**_δ_ = q × m normalized model matrix for dominance effects of SNP markers, **δ** = m × 1 column vector of dominance effects of SNP markers, **a** = **T**_α_**α** = q × 1 genomic breeding values, **d** = **T**_δ_**δ** = q × 1 genomic dominance deviations, **A**_g_ = q × q genomic additive relationship matrix = **T**_α_**T**_α_ ', **D**_g_ = q × q genomic dominance relationship matrix = **T**_δ_**T**_δ_ ', and ,  and  are additive, dominance and residual variances, respectively. The mixed model and its variance-covariance matrix for the QM set of formulations are:
34

where **Z**_1_ = **ZT**_α_ and **Z**_2_ = **ZT**_δ_. Computing difficulty is the **V**^−1^ and **P** = **V**^−1^ − **V**^−1^**X**(**X**’**V**^−1^**X**)^−^**X**’**V**^−1^ for the CE set of Equations 1–2 and is the inverse of the coefficient matrix of the mixed model equations after absorbing fixed non-genetic effects (to be denoted by **C**^−1^) for the QM set of Equations 3–4. The CE set has the best potential for using large numbers of SNP markers because the size of the **V**^−1^ and **P** matrices is determined by the number of individuals (assuming one observation per individual) and does not change for different numbers of SNPs. Similarly, the QM set has the best potential for using large numbers of individuals because the size of the **C**^−1^ matrix is determined by the number of SNP markers and does not change for different numbers of individuals.

### EM-REML and AI-REML

Two algorithms for restricted maximum likelihood (REML) estimation of variance components are implemented in both GREML_CE and GREML_QM: EM type algorithm (EM-REML) and AI-REML algorithm [5]. AI-REML generally is much faster than EM-REML but is not as robust as EM-REML and may be sensitive to initial values of variance components in the iterations. We require at least two iterations of EM-REML and the user may specify a larger number of EM-REML iterations to produce better initial values of variance components than the user provided initial values before switching to AI-REML. When AI-REML yields a negative estimate for any of the variance component estimates, the program automatically returns to EM-REML, which yields non-negative estimates of variance components. This strategy is designed to guarantee GREML_CE and GREML_QM estimates of variance components to be positive.

### Shared memory parallel computing

GVCBLUP is programmed in C++ language using Eigen [16] and Intel Math Kernel libraries (MKL) [17]. Eigen is a C++ template library for linear algebra, supports large dense and sparse matrices and supplies easy-to-use coding expression for linear algebra. Intel MKL provides BLAS and LAPACK linear algebra routines and is optimized for Intel processors with multiple cores by using shared memory parallel computing technology, which is used for dense matrix inversion including **V**^−1^ and **C**^−1^ as well as dense matrix multiplications involving those two matrices in GVCBLUP.

### Calculation and graphical viewing of SNP effects and heritabilities

Both GREML_CE or GREML_QM can output additive and dominance marker effects as well as additive and dominance marker heritabilities for every SNP. SNP additive and dominance effects for GREML_CE are calculated at the last GREML iteration using the following formulations:
56

where  = GBLUP of SNP average effects of gene substitution,  = GBLUP of SNP dominance effects, **P** = **V**^−1^ − **V**^−1^**X**(**X**’**V**^−1^**X**)^−^**X**’**V**^−1^, and where **V** is defined by Equation 2. SNP effects for GREML_QM are obtained directly from the mixed model equations for the QM model (Equation 19 in [5]). According to the EM-REML formulation of additive or dominance variance component [5], we calculate the variance of each SNP marker as the marker contribution to the whole-genome SNP variance defined by its EM-REML formula. Let  = additive variance of the i_th_ SNP, and  = dominance variance of the i_th_ SNP. Then, for GREML_CE, additive and dominance variances of the i_th_ SNP are calculated as:


and for GREML_QM,


where  = additive GBLUP of the i_th_ SNP,  = dominance GBLUP of the i_th_ SNP, r = rank of the coefficient matrix of the mixed model equations, , , , and **C**^αα^ and **C**^δδ^ are defined by Equation 22 in [5]. For the i_th_ SNP marker, additive heritability or heritability in the narrow sense (), dominance heritability () and the total heritability or heritability in the broad sense () are:
789

where  = phenotypic variance,  = total additive heritability of all SNP markers, and  = total dominance heritability of all SNP markers. The output file for the SNP effects and heritabilities of Equations 5-9 is designed such that the SNP effects and heritability estimates can be directly used as the input file for graphing and graphical viewing by SNPEVG2 [18].

### Simulated test data

Two simulated datasets are supplied in GVCBLUP package for testing purpose. One data set (dataset_1) has 1000 genotyped individuals with 3000 SNP markers and the other (dataset_2) has 3000 genotyped individuals with 1000 SNP markers. The parameter files to run GVCBLUP programs for the simulated datasets are also included in the package. These simulated data are designed for GVCBLUP exercises and for showing the complementary advantages of the CE and QM sets of formulations. Users interested in GVCBLUP exercises using large datasets could use a publically available swine dataset with over 45,000 SNP markers on 3534 individuals [19] that was used for comparing GREML estimates by GVCBLUP with the corresponding REML estimates using pedigree relations [5].

### Results and discussion

**Figure 1:**
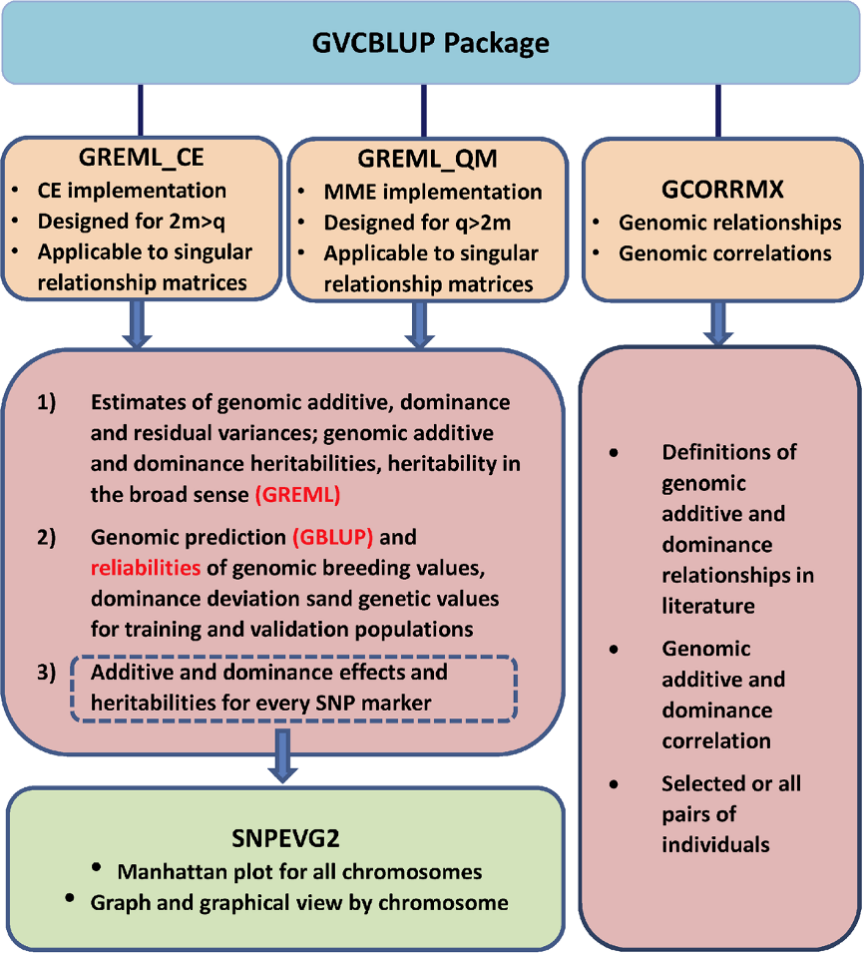
**Structure of the GVCBLUP package.** (m = number of SNP markers, q = number of individuals).

The structure of the GVCBLUP package with three main programs of GREML_CE, GREML_QM and GCORRMX is shown in Figure 1, and details of each program are described below.

### GREML_CE and GREML_QM programs

**Figure 2:**
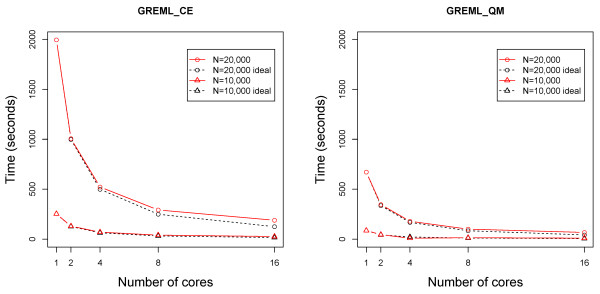
**Excellent scalability of shared memory parallel computing of GREML_CE (left) and GREML_QM (right).**

The GREML_CE and GREML_QM programs calculate GREML estimates of additive, dominance and residual variances, additive and dominance heritabilities, as well as heritability in the broad sense as the summation of the additive and dominance heritabilities. GBLUP and reliability of breeding value, dominance deviation and genotypic value (summation of breeding value and dominance deviation) of each individual in the training or validation population are calculated at the end of variance component estimation. GREML_CE and GREML_QM offer complementary computing advantages with identical GREML and GBLUP results: GREML_CE for large numbers of SNP markers and GREML_QM for large numbers of individuals. Assuming one observation per individuals, GREML_CE is more efficient than GREML_QM if 2 m > q and is less efficient than GREML_QM if q > 2 m, where q = number of individuals and m = number of SNP markers. The example in Table 1 shows the complementary computing advantages of GREML_CE and GREML_QM. Both programs produced identical results (Additional file 1: Supplementary output file) and required the same numbers of iterations (Table 1). For 1000 individuals and 3000 SNP markers, GREML_CE required 5 seconds and GREML_QM required 69 seconds, whereas for 3000 individuals and 1000 SNP markers, GREML_CE required 32 seconds and GREML_QM required 6 seconds (Table 1). Given q = 2 m, the required memory storage of GREML_QM is approximately 1.5 times larger than GREML_CE, but GREML_QM is faster than GREML_CE due to the fact that GREML_CE requires twice as many matrix multiplication between large dense matrices. The shared memory parallel computing of GREML_CE and GREML_QM achieved excellent scalability on ItascaSB cluster with two eight-core Sandy bridge E5-2670 processor chips (2.6 GHz) per node, 256 Gb memory, and Linux operating system (Figure 2). Scalability refers to the stability of average performance of a parallel program as the number of processors increases. Ideal scalability is achieved when the efficiency of k processor-cores (E_k_) is E_k_ = S_k_/k = 1, where S_k_ = the ratio of the execution time with one processor-core to the execution time of the parallel algorithm with k processor-cores [20].

**Table 1:** **Computing time (seconds) using GREML_CE and GREML_QM for simulated datasets** ^**1**^

	q = 1000, m = 3000 (Dataset_1)		q = 3000, m = 1000 (Dataset_2)	
	GREML_CE	GREML_QM	GREML_CE	GREML_QM
Time for SNP input, **A**_g_ and **D**_g_	1	1	1	1
Time per iteration	~0.2	6	3	~0.6
Number of iteration	10	10	7	7
Total time	5	69	32	6

^1^The two programs were run on a personal computer (PC) with Intel Core i7-2600 (4 cores) of 3.40 GHz and memory of 8 Gb.

**Figure 3:**
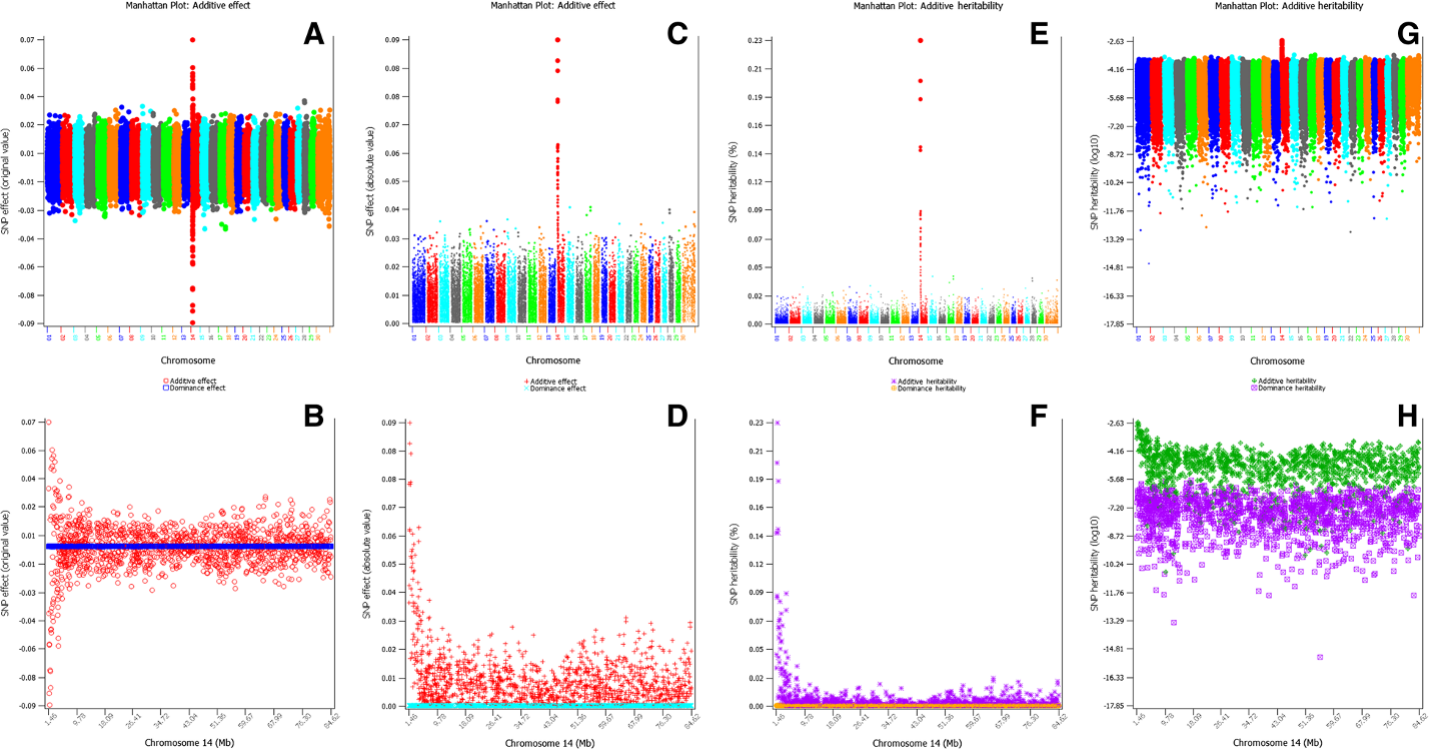
**Graphical viewing of SNP additive and dominance effects and heritabilities. A**: Manhattan plot of the original GBLUP values of SNP additive effects. **B**: Chromosome 14 graph of the original GBLUP values of SNP additive and dominance effects. **C**: Manhattan plot of the absolute GBLUP values of SNP additive effects. **D**: Chromosome 14 graph of the absolute GBLUP values of SNP additive and dominance effects. **E**: Manhattan plot of SNP additive heritabilities in percentage scale. **F**: Chromosome 14 graph of SNP additive and dominance heritabilities in percentage scale. **G**: Manhattan plot of SNP additive heritabilities in log_10_ scale. **H**: Chromosome 14 graph of SNP additive and dominance heritabilities in log_10_ scale. Dominance GBLUP values were all virtually zero, consistent with the fact that the phenotypic values for fat percentage were PTA values of additive effects. The highly significant chromosome 14 region is the *DGAT1* region, and the graphs of C-F are similar to those using stratification corrections reported in Ma *et al*. [21]. The total additive heritability of SNP markers in the 1675278–4606904 Mb region of chromosome 14 that includes *DGAT1* was 0.0248. Although additive heritabilities of other SNPs were much smaller than those in and near the *DGAT1* region, those additive heritabilities were still considerably larger than dominance heritabilities, which were all virtually zero for all SNPs.

**Figure 4:**
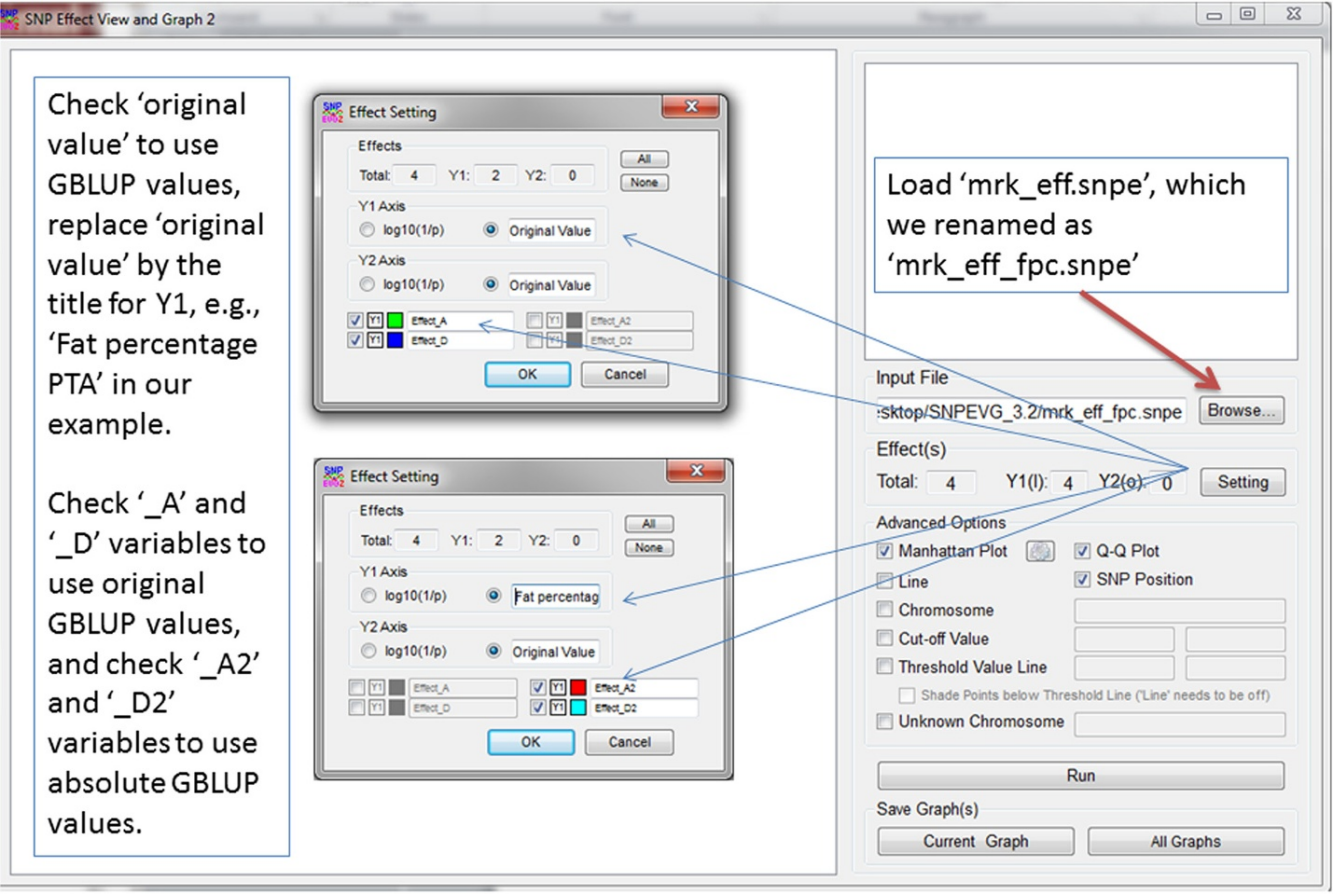
**Procedure of using SNPEVG2 to generate graphs and interactive graphical views.** This procedure can be summarized as: 1) Open SNPEVG2, 2) Load the ‘mark_effect.snpe’ file using ‘Browse’ tab on the GUI of SNPEVG2, 3) click ‘Setting’ and check ‘original value’ for Y1 axis, 4) change ‘original value’ to user defined title for Y1 axis, 5) Click the button pointed by the green arrow to define pixel size and to select color template for the graphs, 6) Click ‘run’, 7) View the graph by scrolling up and down in the top right window, 8) Save ‘All graphs’ or ‘Current graph’. SNPEVG2 is included in the SNPEVG package that is freely available at: http://animalgene.umn.edu/.

GREML_CE and GREML_QM each has three output files for results of GREML, GBLUP, and SNP effects and heritabilities, in addition to screen displays (Additional file 1: Supplementary output files). The GREML output files contain estimates and standard errors of variance components at each iteration, and the final estimates of variance components, heritabilities and their standard errors. The GBLUP output file contains GBLUP of breeding values, dominance deviations, genotypic values, and the corresponding reliabilities for both training and validation populations. These GBLUP results are calculated using the GREML estimates at the last iteration. Both GREML_CE and GREML_QM have a user option to output SNP additive and dominance marker effects and heritbilities for every SNP. The SNP effects and heritabilities can be readily graphed and displayed by SNPEVEG2 [18] including Manhattan plots and graphs by chromosome using the original SNP GBLUP values (Figure 3: A and B), or the absolute SNP GBLUP values (Figure 3: C and D), or SNP additive and dominance heritabilities in the scale of percentages (Figure 3: E and F), or SNP additive and dominance heritabilities in the log_10_ scale (Figure 3: G and H). The procedure to generate the Manhattan plots and chromosome figures is shown in Figure 4.

Numerical evaluations showed that the AI-REML algorithm for both GREML_CE and GREML_QM had fast convergence rate, requiring between 12–20 iterations to converge with a strict tolerance level of 10^−8^, compared to 295–458 iterations using EM-REML (Table 2). The SNP input and the calculation of genomic relationships matrices (**A**_g_ and **D**_g_) required more computing time than per-iteration of the estimation step. GREML_CE was able to use 50,000 individuals with 400 K SNP markers with total computing time about 23 hours for 13 iterations. For 20,000 individuals and one million SNP markers, GREML_CE only required 4.8 hours. GREML_QM was highly efficient for using low-density SNP markers, requiring only 2 hours for 200,000 individuals with 10 K SNP markers. For 100,000 individuals with 50 K SNP markers, GREML_QM required about 46 hours for 20 iterations (Table 2). Although AI-REML was fast, extreme heritability levels (0 or 1) generally would cause failure of AI-REML. For eight of ten replications with null heritability, AI-REML failed, but the variance components still could be estimated with EM-REML (Table 3). AI-REML was successful for all ten replications with heritability of 0.3.

**Table 2:** **Capacity and speed of GVCBLUP for genomic estimation of additive, dominance and residual variances (tolerance = 10** ^**−8**^ **) and ItascaSB supercomputer**

	GREML_CE	GREML_CE	GREML_QM	GREML_QM ^1^
Number of individuals (q)	20,000	50,000	200,000	100,000
Number of SNP markers (m)	1 million	400,000	10,000	50,000
Time for SNP input, A_g_ and D_g_	3.7 hrs	6.0 hrs	14.9 min	0.33 hrs
Time per iteration	3.1 min	0.77 hrs	1.5 min	2.25 hrs
Total time	4.8 hrs	23.2 hrs	2 hrs	~45.83 hrs
Number of iteration	12	13	20	20

^1^Computing time for calculating GBLUP reliabilities is not included.

**Table 3:** **Comparison of iteration numbers of EM-REML and AI-REML (tolerance = 10** ^**−8**^ **) using simulated data with different heritability levels**

Replication	***h***_***α***_^2^ = 0.0, ***h***_***δ***_^2^ = 0.0		***h***_***α***_^2^ = 0.3, ***h***_***δ***_^2^ = 0.3	
	EM-REML	AI-REML	EM-REML	AI-REML
1	173	−^1^	322	9
2	231	-	386	12
3	348	-	348	9
4	359	-	354	8
5	481	18	458	10
6	138	-	295	10
7	871	-	416	8
8	134	-	353	9
9	291	16	336	12
10	1000	1000^1^	431	11

^1^AI-REML failed.

In addition to the tests in Table 1 using the simulation datasets we provide with the GVCBLUP package, GREML_CE and GREML_QM programs were extensively evaluated using simulation data under various assumptions, and the GREML estimates were compared to the REML estimates of additive heritabilities of five traits using pedigree relationships in a publically available swine dataset of 3534 pigs with the 60 K SNP data [5]. GREML and GBLUP generally were able to capture small additive and dominance effects that each accounted for 0.00005-0.0003 of the phenotypic variance and GREML was able to differentiate true additive and dominance heritability levels [5]. The inclusion of dominance in the prediction model resulted in improved accuracy of genomic prediction [8], and the genomic models with additive and dominance effects were more accurate for the estimation of variance components than their pedigree-based counterparts [7]. In a study of trout propensity to migrate, genomic-predicted additive effects completely separated migratory and nonmigratory fish in the wild population with 95.5% additive heritability and 4.5% dominance heritability, whereas genomic-predicted dominance effects achieved such complete separation in the dam-blocked population with 0% additive heritability and 39.3% dominance heritability [22], showing the importance to account for the exact effect type in the prediction model.

### GCORRMX program

The GCORRMX program is designed to calculate measures of genomic similarities among individuals. This program currently calculates the **A**_*g*_ and **D**_*g*_ matrices for six definitions [23]. An example of the GCORRMX output files is given in Additional file 1: Supplementary output files.

### GVCeasy: Graphical user interface (GUI) for GVCBLUP

**Figure 5:**
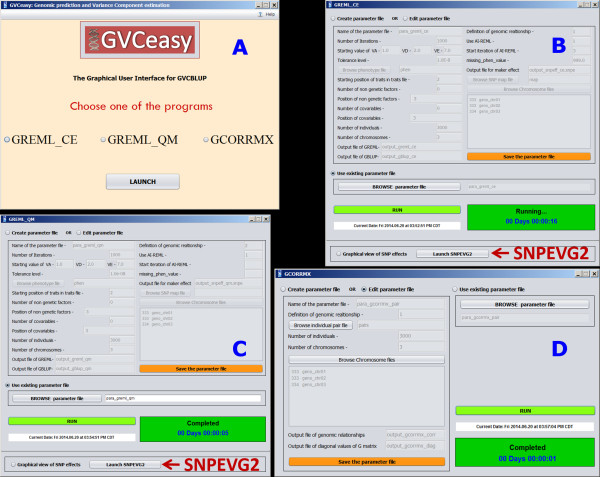
**GVCeasy graphical user interface (GUI) for GVCBLUP. A**: The main control of GVCeasy. Any of the three main programs may be launched from here and the same program may be opened multiple times. **B**: The GUI for GREML_CE with a tab to lunch SNPEVG2 to graph and view SNP additive and dominance effects. **C**: The GUI for GREML_QM with a tab to lunch SNPEVG2 to graph and view SNP additive and dominance effects. **D**: The GUI for GCORRMX.

The three main programs of GVCBLUP are command line programs. GVCeasy is a Java program developed as a user-friendly GUI with a capability to run GVCBLUP by mouse clicks, providing considerable convenience for users not familiar with command line operations. GVCeasy can lunch any of the three main programs of GVCBLUP and provides a capability of editing the parameter file for each main program (Figure 5). In addition, SNPEVG2 can be launched from the GREML_CE or GREML_QM window of GVCeasy for graphical viewing of SNP additive and dominance effects. To run GVCeasy, the programs of GVCeasy, GREML_CE, GREML_QM, GCORRMX and the SNPEVG package that includes SNPEVG2 need to be placed in the same directory. GVCeasy is applicable to Windows, Linux and Mac OS X versions of GVCBLUP.

## Conclusions

The GVCBLUP package is a powerful and user friendly computing tool for assessing the type and magnitude of genetic effects affecting a phenotype by estimating whole-genome additive and dominance heritabilities of a phenotype using genome-wide SNP markers, is a full featured computing tool for genomic prediction of breeding values, dominance deviations and genotypic values for both training and validation data sets, and provides an important computing utility for research and education in the area of genomic prediction and estimation.

## Availability and requirements

**Project name**: GVCBLUP

**Project home page:**http://animalgene.umn.edu/

**Operating system(s)**: Windows, Linux and Mac OS X

**Programming language:** C++, Java

**License:** None

## Electronic supplementary material

Additional file 1: **Supplementary output files.** (PDF 42 KB)

## Abbreviations

SNP: Single nucleotide polymorphism
BLUP: Best unbiased linear prediction
GBLUP: Genomic BLUP
REML: Restricted maximum likelihood estimation
GREML: Genomic REML
EM: Expectation-maximization
AI-REML: Average information REML
GUI: Graphical user interface
MME: Mixed model equations.
