# Supplementary material for: GVCBLUP: a computer package for genomic prediction and variance component estimation of additive and dominance effects
Source: BMC Bioinformatics. 2014 Aug 9;15(1):270. doi: 10.1186/1471-2105-15-270 (PMC4133608; doi:10.1186/1471-2105-15-270)
Supplement: Supplementary file 1 — Additional file 1: Supplementary output files. (PDF 42 KB) [file 12859_2014_6536_MOESM1_ESM.pdf]

Additional file 1: Supplementary output files

The four GREML output files (Files 1-4) below are GREML estimates by GREML_CE and GREML_QM for the two test data sets. Files 1 and 2 use the dataset with 3000 SNP markers and 1000 individuals, and Files 3 and 4 use the dataset with 1000 SNP markers and 3000 individuals. In addition to showing GREML estimates from GVCBLUP, these four files are numerical examples in support the following conclusions:

1) GREML_CE and GREML_QM have identical results (same results at every iteration and the same number of iterations), as shown by the comparison between File 1 and File 2, and between File 3 and File 4;
2) GREML_CE and GREML_QM have complementary computing advantages, as shown by the comparison between File 1 and File 3, and between File 2 and File 4.

File 5 is an example of the output file of the GCORRMX program.

File 1: Estimates of variance components and heritabilities by GREML_ CE for 3000 SNP markers and 1000 individuals (file name: output_greml_ce)

```
Output of GREML results from GREML_CE.

Number of markers: 3000
Number of chromosomes: 3

Number of phenotypic observations: 1000
  Number of individuals with phenotypic observations: 1000
  Number of individuals without phenotypic observations: 0

Iteration VA             Tolerance_VA   VD             Tolerance_VD   VE             Tolerance_VE
1         4.706959e-001  5.293041e-001  6.244678e-001  1.375532e+000  1.629727e+000  5.370273e+000
2         6.567722e-001  1.860762e-001  6.919474e-001  6.747955e-002  1.426925e+000  2.028014e-001
3         9.783415e-001  3.215693e-001  1.133466e+000  4.415185e-001  8.480809e-001  5.788443e-001
4         1.103954e+000  1.256125e-001  1.101448e+000  3.201764e-002  9.287548e-001  8.067394e-002
5         1.128407e+000  2.445282e-002  1.093826e+000  7.622571e-003  9.343307e-001  5.575851e-003
6         1.131210e+000  2.803324e-003  1.093079e+000  7.465113e-004  9.341486e-001  1.820620e-004
7         1.131519e+000  3.087446e-004  1.092976e+000  1.028650e-004  9.341331e-001  1.550429e-005
8         1.131554e+000  3.463923e-005  1.092965e+000  1.134782e-005  9.341312e-001  1.924986e-006
9         1.131557e+000  3.878090e-006  1.092964e+000  1.277036e-006  9.341310e-001  2.124590e-007
10        1.131558e+000  4.343798e-007  1.092964e+000  1.429360e-007  9.341310e-001  2.387022e-008
```

```
11        1.131558e+000  4.865057e-008  1.092964e+000  1.601138e-008  9.341310e-001  2.672083e-009
12        1.131558e+000  5.448952e-009  1.092964e+000  1.793256e-009  9.341310e-001  2.993082e-010
SE        2.070319e-001                 1.492317e-001                 9.426440e-002


Inverse of AI matrix:
  4.286222e-002 -6.019587e-003 -2.955132e-003
 -6.019587e-003  2.227011e-002 -7.730389e-003
 -2.955132e-003 -7.730389e-003  8.885777e-003

Additive heritability, SE           : 3.582407e-001, 4.883500e-002
Dominance heritability, SE          : 3.460221e-001, 4.613524e-002
Heritability in the broad sense, SE : 7.042628e-001, 3.678706e-002

Total running time: 0 days 0 hours 0 minutes 5 seconds.
```

File 2: Estimates of variance components and heritabilities by GREML_ QM for 3000 SNP markers and 1000 individuals (file name: output_greml_qm)

```
Output of GREML results from GREML_QM.

Number of markers: 3000
Number of chromosomes: 3

Number of phenotypic observations: 1000
  Number of individuals with phenotypic observations: 1000
  Number of individuals without phenotypic observations: 0

Iteration VA             Tolerance_VA   VD             Tolerance_VD   VE             Tolerance_VE
1         4.706959e-001  5.293041e-001  6.244678e-001  1.375532e+000  1.629727e+000  5.370273e+000
2         6.567722e-001  1.860762e-001  6.919474e-001  6.747955e-002  1.426925e+000  2.028014e-001
3         9.783415e-001  3.215693e-001  1.133466e+000  4.415185e-001  8.480809e-001  5.788443e-001
4         1.103954e+000  1.256125e-001  1.101448e+000  3.201764e-002  9.287548e-001  8.067394e-002
5         1.128407e+000  2.445282e-002  1.093826e+000  7.622571e-003  9.343307e-001  5.575851e-003
6         1.131210e+000  2.803324e-003  1.093079e+000  7.465113e-004  9.341486e-001  1.820620e-004
7         1.131519e+000  3.087446e-004  1.092976e+000  1.028650e-004  9.341331e-001  1.550429e-005
8         1.131554e+000  3.463923e-005  1.092965e+000  1.134782e-005  9.341312e-001  1.924986e-006
9         1.131557e+000  3.878090e-006  1.092964e+000  1.277036e-006  9.341310e-001  2.124590e-007
10        1.131558e+000  4.343798e-007  1.092964e+000  1.429360e-007  9.341310e-001  2.387022e-008
```

```
11        1.131558e+000  4.865058e-008  1.092964e+000  1.601137e-008  9.341310e-001  2.672094e-009
12        1.131558e+000  5.448938e-009  1.092964e+000  1.793271e-009  9.341310e-001  2.992959e-010
SE        2.070319e-001                 1.492317e-001                 9.426440e-002


Inverse of AI matrix:
  4.286222e-002 -6.019587e-003 -2.955132e-003
 -6.019587e-003  2.227011e-002 -7.730389e-003
 -2.955132e-003 -7.730389e-003  8.885777e-003

Additive heritability, SE           : 3.582407e-001, 4.883500e-002
Dominance heritability, SE          : 3.460221e-001, 4.613524e-002
Heritability in the broad sense, SE : 7.042628e-001, 3.678706e-002

Total running time: 0 days 0 hours 1 minutes 19 seconds.
```

File 3: Estimates of variance components and heritabilities by GREML_ CE for 1000 SNP markers and 3000 individuals (file name: output_greml_ce)

```
Output of GREML results from GREML_CE.

Number of markers: 1000
Number of chromosomes: 3

Number of phenotypic observations: 3000
  Number of individuals with phenotypic observations: 3000
  Number of individuals without phenotypic observations: 0

Iteration VA             Tolerance_VA   VD             Tolerance_VD   VE             Tolerance_VE
1         6.138855e-001  3.861145e-001  7.062130e-001  1.293787e+000  1.272038e+000  5.727962e+000
2         8.448634e-001  2.309779e-001  8.718213e-001  1.656083e-001  1.090407e+000  1.816303e-001
3         1.052127e+000  2.072634e-001  1.053719e+000  1.818977e-001  9.855451e-001  1.048622e-001
4         1.095827e+000  4.369979e-002  1.074924e+000  2.120526e-002  9.902820e-001  4.736905e-003
5         1.097768e+000  1.941963e-003  1.075502e+000  5.776256e-004  9.902018e-001  8.017934e-005
6         1.097788e+000  1.923879e-005  1.075513e+000  1.091585e-005  9.901979e-001  3.903467e-006
7         1.097788e+000  3.272541e-007  1.075513e+000  1.004536e-007  9.901978e-001  5.073248e-008
8         1.097788e+000  3.679218e-009  1.075513e+000  1.921815e-009  9.901978e-001  7.512063e-010
SE        1.402329e-001                 8.048602e-002                 3.230763e-002
```

```
Inverse of AI matrix:
  1.966526e-002 -3.194976e-004 -6.312981e-004
 -3.194976e-004  6.477999e-003 -4.945340e-004
 -6.312981e-004 -4.945340e-004  1.043783e-003

Additive heritability, SE           : 3.470171e-001, 3.097606e-002
Dominance heritability, SE          : 3.399758e-001, 2.330417e-002
Heritability in the broad sense, SE : 6.869928e-001, 1.863563e-002

Total running time: 0 days 0 hours 0 minutes 32 seconds.
```

File 4: Estimates of variance components and heritabilities by GREML_ QM for 1000 SNP markers and 3000 individuals (file name: output_greml_qm)

```
Output of GREML results from GREML_QM.

Number of markers: 1000
Number of chromosomes: 3

Number of phenotypic observations: 3000
  Number of individuals with phenotypic observations: 3000
  Number of individuals without phenotypic observations: 0


Iteration VA             Tolerance_VA   VD             Tolerance_VD   VE             Tolerance_VE
1         6.138855e-001  3.861145e-001  7.062130e-001  1.293787e+000  1.272038e+000  5.727962e+000
2         8.448634e-001  2.309779e-001  8.718213e-001  1.656083e-001  1.090407e+000  1.816303e-001
3         1.052127e+000  2.072634e-001  1.053719e+000  1.818977e-001  9.855451e-001  1.048622e-001
4         1.095827e+000  4.369979e-002  1.074924e+000  2.120526e-002  9.902820e-001  4.736905e-003
5         1.097768e+000  1.941963e-003  1.075502e+000  5.776256e-004  9.902018e-001  8.017934e-005
6         1.097788e+000  1.923879e-005  1.075513e+000  1.091585e-005  9.901979e-001  3.903467e-006
7         1.097788e+000  3.272541e-007  1.075513e+000  1.004536e-007  9.901978e-001  5.073248e-008
8         1.097788e+000  3.679212e-009  1.075513e+000  1.921814e-009  9.901978e-001  7.512053e-010
SE        1.402329e-001                 8.048602e-002                 3.230763e-002
```

```
Inverse of AI matrix:
  1.966526e-002 -3.194976e-004 -6.312981e-004
 -3.194976e-004  6.477999e-003 -4.945340e-004
 -6.312981e-004 -4.945340e-004  1.043783e-003

Additive heritability, SE           : 3.470171e-001, 3.097606e-002
Dominance heritability, SE          : 3.399758e-001, 2.330417e-002
Heritability in the broad sense, SE : 6.869928e-001, 1.863563e-002

Total running time: 0 days 0 hours 0 minutes 6 seconds.
```

File 5: Example of the output file of GCORRMX for calculating genomic relationships (file name: output_greml_qm)

```
GCORRMX:  Definition I for additive and dominance relationships
               Ind_1               Ind_2       Additive      Dominance
                2121                2122    5.8511e-001    2.4373e-001
                2121                2123    5.1428e-001    3.1196e-001
                2121                2124    4.7795e-001    3.5445e-001
                2121                2125    2.6133e-001   -2.2135e-002
                2121                2126    7.1610e-001    5.2187e-001
                2121                2127    2.6133e-001   -2.2135e-002
                2121                2128    5.3774e-001    3.5330e-001
                2121                2129    8.4005e-001    7.5453e-001
                2122                2123    6.3007e-001    2.5615e-001
                2122                2124    5.9785e-001    1.8100e-001
                2122                2125    6.6044e-001    3.1495e-001
                2122                2126    8.4215e-001    7.2798e-001
                2122                2127    6.6044e-001    3.1495e-001
                2122                2128    3.7226e-001    1.0287e-002
                2122                2129    7.2590e-001    4.8420e-001
                2123                2124    9.4378e-001    8.9339e-001
                2123                2125    7.5385e-001    5.0227e-001
                2123                2126    7.6106e-001    5.3429e-001
                2123                2127    7.5385e-001    5.0227e-001
                2123                2128    2.8295e-001    7.2152e-002
                2123                2129    6.4480e-001    5.0274e-001
                2124                2125    7.9554e-001    5.7780e-001
                2124                2126    7.2884e-001    4.5913e-001
                2124                2127    7.9554e-001    5.7780e-001
```

2124	2128	3.7186e-001	1.7851e-001
2124	2129	6.1258e-001	4.2758e-001
2125	2126	5.0195e-001	3.4453e-004
2125	2127	1.0511e+000	1.0822e+000
2125	2128	2.7637e-001	2.3282e-001
2125	2129	3.8570e-001	-3.1209e-002
2126	2127	5.0195e-001	3.4453e-004
2126	2128	4.9915e-001	7.1613e-002
2126	2129	8.5689e-001	7.6234e-001
2127	2128	2.7637e-001	2.3282e-001
2127	2129	3.8570e-001	-3.1209e-002
2128	2129	6.2309e-001	3.0428e-001
